# Fungal Periprosthetic Hip Joint Infections

**DOI:** 10.3390/diagnostics12102341

**Published:** 2022-09-27

**Authors:** Christos Koutserimpas, Symeon Naoum, Vasileios Giovanoulis, Konstantinos Raptis, Kalliopi Alpantaki, Konstantinos Dretakis, Georgia Vrioni, George Samonis

**Affiliations:** 1 Department of Orthopaedics and Traumatology, “251” Hellenic Air Force General Hospital of Athens, 115 25 Athens, Greece; 2 2nd Department of Orthopaedics, “Hygeia” General Hospital of Athens, 151 23 Marousi, Greece; 3 Department of Orthopaedics, Croix Rousse Hospital, University of Lyon, 69004 Lyon, France; 4 Department of Orthopaedics and Traumatology, “Venizeleion” General Hospital of Crete, 714 09 Iraklio, Greece; 5 Department of Microbiology, Medical School, National and Kapodistrian University of Athens, 115 27 Athens, Greece; 6 Department of Medicine, University of Crete, 715 00 Heraklion, Greece; 7 First Department of Medical Oncology, “Metropolitan” Hospital, Neon Faliron, 185 47 Attica, Greece; 8 Department of Internal Medicine, University Hospital of Heraklion, 715 00 Heraklion, Greece

**Keywords:** fungal hip infection, *Candida* hip infection, total hip arthroplasty infection, *Aspergillus* hip infection, hip arthroplasty complications

## Abstract

**Introduction:** Fungal hip prosthetic joint infections (PJIs) are rare but severe infections. Their incidence has increased in the last decades due to the aging population, as well as due to the increased number of immunosuppressed hosts. The present review of all published fungal PJIs in hip arthroplasties aims to present as much data as possible for both medical and surgical treatment options, so that the best applicable management may be concluded. **Methods:** A meticulous review of all published fungal hip PJIs was conducted. Information regarding demographics, causative fungus, antifungal treatment (AFT), surgical management as well as the infection outcome was recorded. **Results:** A total of 89 patients suffering fungal hip PJI were identified. The patients’ mean age was 66.9 years. The mean time from initial arthroplasty to onset of symptoms was 69.3 months, while 40.4% of the patients were immunocompromised. The most common imaging method indicating diagnosis was plain X-ray or CT scan (20.2%), while definite diagnosis had become possible through cultures in most cases (98.9%), and/or histology (44.9%). The most frequently isolated fungus was *C. albicans* (49.4%), followed by *C. parapsilosis* (18%) and *C. glabrata* (12.4%), while bacterial co-infection was present in 32 cases (36%). Two-stage revision arthroplasty (TSRA) was the most commonly performed procedure (52.8%), with mean time between the two stages = 7.9 months. Regarding antifungal treatment (AFT), fluconazole was the preferred agent (62.9%), followed by amphotericin B (36%), while the mean duration of AFT was 5.1 months. Outcome was successful in 68 cases (76.4%). **Conclusions:** Both diagnosis and management of fungal PJIs in patients having undergone total hip arthroplasty are quite demanding. A multidisciplinary approach is of utmost importance, since the combination of AFT and TSRA appears to be the proper treatment method.

## 1. Introduction

Total hip arthroplasty (THA) represents a successful, widely performed orthopedic procedure for patients with a variety of painful conditions regarding the hip joint. THA may alleviate pain and restore function as well as improve quality of life. Approximately 500,000 THAs are being conducted each year in the United States [1].

Joint reconstruction surgery has greatly improved over the years, as minimally invasive surgical approaches and better postoperative pain management and blood transfusion reduction protocols have been developed, as well as that there is outstanding improvement in navigation, robotic surgery and new prosthetic materials [2,3,4,5].

As life expectancy and the number of THAs have been widely extended, revision reconstruction surgery rates have also been increased [6]. Revision surgery is a very challenging procedure, with a higher risk of complications and variety of success rates [6]. The surgeon faces technical challenges regarding surgical approach and loss of host bone stock as well as the removal of the primary implant [6,7]. The main causes of prosthetic failure encompass infection, aseptic loosening and periprosthetic fractures [5,8].

Prosthetic joint infections (PJIs) regarding THA are considered rare complications, with an incidence of 0.5–1.0%. However, they represent a catastrophic complication regarding quality of the patient’s life, while, occasionally, they may even prove to be lethal [9]. Risk factors for PJI include prolonged duration of surgery, postoperative complications such hematoma and wound dehiscence, tourniquet time and cement type, and comorbidities such as diabetes, malignancy, chronic kidney disease, obesity, immunosuppression and the increased American Society of Anesthesiologists (ASA) grade as well as blood transfusion requirement have been reported [10].

In particular, regarding microorganisms responsible for PJIs, fungi are relatively rare causative organisms, reported to be cultured in about 1–2% of all cases. This incidence has increased in the last decades due to an aging population, as well as to the increased number of immunosuppressed hosts [11,12,13]. Although no official guidelines have been established for fungal PJIs, currently, based on limited data, a two-stage revision arthroplasty (TSRA) combined with long-term antifungal treatment (AFT) is recommended [11,14].

The present study represents an effort, by reviewing all published fungal PJI cases in THAs, to collect as much data as possible for both medical and surgical treatment options so that the best applicable management may be concluded.

## 2. Materials and Methods

A meticulous electronic search of PubMed and MEDLINE databases was conducted so that all existing articles regarding cases of fungal PJIs occurring in THAs were selected. The study period was from January 2000 to May 2022. Alone and/or in combination, the terms “fungal infection”, “periprosthetic joint infection”, “total hip replacement infection”, “total hip arthroplasty infection”, “*Candida* periprosthetic joint infection”, “*Aspergillus* periprosthetic joint infection”, “Coccidioidal periprosthetic joint infection”, “mold hip infection”, “yeast hip infection” and “fungal arthroplasty infection” were used. Following the identification of these cases, individual references listed in each publication were further investigated for ascertainment of additional cases.

The present review was limited to papers published in English and in peer-reviewed journals. Expert opinions, book chapters, studies on animals, on cadavers or in vitro investigations, as well as abstracts in scientific meetings were excluded. Additionally, studies without information about the specific antifungal agent used during management were not included.

The data extracted from these studies included age, gender, country of origin, the presence of immunosuppressive conditions/comorbidities, the previous use of antimicrobials, the presence of bacterial co-infection, time interval from joint implantation to symptom onset and from symptom to diagnosis, number of previous revisions in the same joint, reason of previous revision surgery, duration and type of AFT as well as the type of surgical intervention. Furthermore, the results of medical and surgical treatment, along with the follow-up of each case, were recorded and evaluated.

Treatment was considered successful if all signs and symptoms of the infection had disappeared and no recurrence was observed during the follow-up period.

Data were recorded and analyzed using Microsoft Excel 2019 (Microsoft Corporation, Redmond, WA, USA).

## 3. Results

A total of 89 patients (45; 50.6% males) suffering fungal PJI occurring in the hip joint, covering a 22-year period, were identified [15,16,17,18,19,20,21,22,23,24,25,26,27,28,29,30,31,32,33,34,35,36,37,38,39,40,41,42,43,44,45,46,47,48,49,50,51,52]. The studied population’s mean age was 66.9 years (standard deviation (SD) = 13.2).

Table 1 depicts the main characteristics of these cases. Most cases were reported from the USA (24; 27%), followed by cases from Germany (20; 22.5%), from China (11; 12.4%), as well as from many other countries around the globe (Table 1).

The mean time from initial arthroplasty implantation surgery to symptom onset was 69.3 months (SD = 132.4), while the mean time from symptom onset to definite diagnosis was 33.8 months (SD = 43.8).

Regarding the causative fungal organisms, the most frequently isolated one was *C. albicans*, found in 44 cases (49.4%), followed by *C. parapsilosis* in 16 (18%), *C. glabrata* in 12 (12.4%), *C. tropicalis* in 5 (5.6%), *C. famata* in 3 (3.3%), *Cryptococcus neoformans* in 2 (2.2%) and *Pseudallescheria boydii, C lipolytica, Aspergillus* spp., *C. lusitaniae*, *Alternaria infectoria*, *Rhodotorula minuta*, *Pithomyces*, *Aureobasidium, Hormonema* and *Coccidioidess* spp. yielded in 1 case each (1.2%).

Bacterial co-infection was present in 32 cases (36%), with the most common microorganism being *Staphylococcus aureus* (8 cases (25%), 5 of them were methicillin-resistant (62.5%)), *S. epidermitis* (4 cases; 12.5%), *Enterococcus faecium* (2; 6.3%), *Streptococcus* spp. (3; 9.4%), *Serratia marcescens* (2; 6.3%)*,* *Pseudomonas aeruginosa* (2; 6.3%) and *E. Coli* (2; 6.3%), other unspecified Gram-negative bacilli (2; 6.3%), and *Citrobacter koser, Staphylococcus capitis*, *Staphylococcus hominis*, *Staphylococcus caprae*, *Staphylococcus haemolyticus*, *Acinetobacter lwoffi* and *Propionibacterium acnes* were cultured in 1 case (3.1%) each.

Detailed information regarding immunosuppressive conditions is exhibited in Table 1. More specifically, 36 patients (40.4%) were suffering at least one potentially immunosuppressive condition, according to the available information from each report. Particularly, 19 patients were suffering diabetes mellitus (53.8%), 7 chronic obstructive pulmonary disease (19.4%), 6 reported chronic steroid usage (16.7%), 5 had a malignancy (13.9%), 5 chronic renal failure (13.9%), 4 rheumatoid arthritis (11.1%), 3 some type of hematological malignancy (8.3%), 3 chronic liver diseases (8.3%), 3 chronic alcohol abuse (8.3%) and 2 hypertension (5.6%), while vasculitis, Sjögren’s syndrome, myasthenia gravis, obesity and systemic lupus erythematosus were reported once each (2.8%).

Regarding previous antibiotic use during the last 6 months from the current infection, in 25 cases (28.1%) administration of an antimicrobial was reported with mean treatment duration = 1.7 months (SD = 1.9). As far as the antibiotic classes are concerned, in 11 cases (44%) Glycopeptides were used, in 6 (24%) Penicillin and Beta-lactamase inhibitors, in 4 (16%) Oxazolidinones, in 3 (12%) Antimycobacterial agents, in 3 (12%) Fluoroquinolones, in 2 (8%) Cephalosporins, in 2 (8%) Carbapenems and Folic Acid inhibitors, and Tetracycline, Penicillin-like antibiotics and Cyclic Lipopeptide were used in 1 (4%) case each. It should also be noted that in 9 cases (36%), although an antibiotic agent was used, the exact class was not reported.

Previous revision reconstruction surgery was reported in 47 cases (52.8%). Regarding the number of previous revisions, the mean value was 3.5 (SD = 2.1), while the most common reasons for revision surgery were infection (23 cases; 50%), aseptic loosening (5; 10.9%) and periprosthetic fracture (1; 2.2%), and in 18 cases (39.1%) the reasons for revision hip surgery were not clarified.

Table 2 highlights diagnostic techniques, including imaging indicating the infection, as well as the methods of firm diagnosis of the disease. Regarding imaging methods indicating diagnosis, plain X-ray or CT scan were performed in 18 patients (20.2%), followed by bone scan in 8 (9%), while magnetic resonance imaging (MRI) was implemented in only 1 case (1.1%; case 27).

Definite diagnosis was possible through periprosthetic tissue and/or joint fluid cultures and histopathology. Moreover, in 88 cases (98.9%), fungal species were cultured (joint fluid in 41 cases, 46.6%, tissue specimen in 77 cases, 87.5%). In 40 cases, fungal PJI was diagnosed through histopathology (44.9%), while serology testing was additionally performed in 1 case (1.1%, case 12). In particular, in 40 cases (38%) fungal PJI was diagnosed through both histopathology and cultures.

Table 3 highlights surgical management and AFT of the reported cases, as well as information regarding time between stages in two-stage revision arthroplasty (TSRA), duration of AFT and outcomes. Regarding surgical treatment, TSRA was the most commonly performed procedure (47; 52.8%), with mean time between the two stages of 7.9 months (SD = 12.5), followed by resection arthroplasty (RA) (19; 21.3%), one-stage revision arthroplasty ((OSRA), 12 cases; 13.5%) and debridement (10; 11.2%), while 4 cases did not receive any surgical treatment (4.5%).

Regarding AFT, 48 cases (53.9%) were treated with one antifungal agent, 33 (37.1%) with two, either simultaneously or consecutively, while 10 (11.2%) were treated with more than two antifungal agents. The mean duration of AFT was found to be 5.1 months (SD = 3.8).

Fluconazole was the preferred agent in 56 cases (62.9%, in 26 (46.4%) as monotherapy), followed by amphotericin B in 32 (36%, in 16 (50%) as monotherapy), caspofungin in 18 (20.2%, 2 (11.1%) as monotherapy), flucytosine in 9 (10.1%, none as monotherapy), voriconazole in 6 (6.7%, in 2 (33.3%) as monotherapy) and micafungin in 2 cases (2.2%, 1 (50%) as monotherapy), while ketoconazole, anidulafungin, posaconazole and miconazole were used in 1 case each (1.1%).

Regarding antimicrobial and/or antifungal regimen (AAMR) in cement, 46 cases (51.7%) were treated with at least one AAMR. In particular, in 17 cases (39.5%) a single AAMR was used, in 13 (30.2%) two, and in 16 (37.2%) more than two. Vancomycin was the agent mostly used (26; 60.5%, 3; 13.6% as monotherapy), followed by amphotericin B (22; 51.2%, 12; 54.5% as monotherapy), gentamycin (22; 51.2%, not as monotherapy), clindamycin (6; 14%, not as monotherapy), fluconazole (4; 9.3%, all as monotherapy) and aztreonam (3; 7%, not as monotherapy), while voriconazole, itraconazole and meropenem were used in 1 case each (2.3%).

During the 2000–2022 period, outcome was successful in 68 cases (76.4%), while the mortality rate was 2.2% (2 cases). The mean follow-up was 44.1 months (SD = 29.7).

## 4. Discussion

The majority of complications related to THA are infrequent and most of them may be prevented if anticipated. However, their significance varies from minor incidents to even fatal situations [53]. The rates of some of these complications may be diminished if proper equipment is available and experienced orthopedic surgeons perform the procedure, in addition to appropriate patient selection, methodical preoperative plan and surgical technique, as well as diligent postoperative treatment [54].

PJI is a serious complication of prosthetic joint implantation and one of the most common reasons for revision surgery [55,56]. PJIs are defined as early onset if they have occurred <3 months after surgery, delayed onset is defined as occurring 3–12 months after surgery and late onset is defined as occurring >12 months after surgery. However, these definitions are controversial and not always absolutely clear, as there is an overlap between early- and delayed-onset PJIs [57]. The incidence of PJI in THA is estimated to be approximately 0.5–1.0%, while bundles of measures have been proposed to eliminate such infections [58,59].

Fungal PJIs’ incidence is approximately 1–2% of all PJIs in total hip arthroplasty cases. Hence, these infections are considered rare. Furthermore, their management, including medical treatment, as well as surgical intervention, is quite challenging [11,12]. It is of note that the management of PJIs may involve several operations, long hospital stays and significant morbidity and mortality [11,12,13].

*Candida* species represent the most common fungus responsible for such PJIs, followed by other fungi [11,12,13]. It is of paramount importance that data regarding AFT, AFT duration and the infections’ outcomes, as well as the kind of surgical interventions, the use of antifungal agents in cement and the time intervals between the two stages of TSRA be analyzed, so that the best application of both medical and surgical therapy may be delivered.

Fungal PJIs are expected to increase, due to the growing rates of prosthetic joint reconstruction, the rise of immunocompromised hosts and the increasing use of invasive devices such as central venous catheters [9,60]. Immunosuppression and systemic diseases have been reported as risk factors for invasive fungal infections [9,61,62]. In the present review, a total of 36 patients (40.4%) were immunocompromised. DM was the most common immunosuppressive factor in these cases (53.8%). Diabetes mellitus is a major risk factor for mycotic infections. Uncontrolled diabetes leads to unfavorable outcomes regarding fungal infection eradication, while anemia, hypoalbuminemia and elevated serum creatinine have been associated with invasive fungal infections in type 2 diabetes patients [63]. DM leads to immunosuppression by impaired innate immunity and acquired immunity. Functions of neutrophils such as phagocyte, chemotaxis and cytokine production are decreased in DM, while hyperglycemia and Th2-axis shift reducing Th1-dependent immunity are observed in DM patients. Nevertheless, the majority of results concerning the interaction of hyperglycemia and immune function are controversial, and the relevance of hyperglycemia and/or hyperinsulinemia to immunosuppressive mechanisms remains unclear [63,64].

Additional risk factors for fungal infections include impaired immune response secondary to rheumatoid arthritis, malignancy, diabetes, malnutrition, indwelling catheters, prolonged antibiotic use and revision arthroplasty surgery [61,62]. It is of note that currently, no guidelines exist for prophylactic AFT in high-risk, immunocompromised hosts undergoing primary or revision hip reconstruction surgery. Nevertheless, taking into account that the number of immunocompromised hosts is increasing, along with the number of patients undergoing THA, it becomes apparent that the rising of such infections will be the subject of future concern and a field of interesting research. Regarding prolonged antibiotic use, antibiotics are a modifiable iatrogenic risk factor for invasive candidiasis, since they alter the normal flora of the host, while other underlying mechanisms remain unclarified [10,14]. In this study, as far as the history of prolonged antibiotic usage is concerned, in 25 cases (28.1%) at least one antibiotic agent had been administered prior to the current infection, with mean duration of 1.7 months.

In the present study, the mean time interval, in all published fungal PJIs in THAs, between initial joint reconstruction surgery and symptom onset was 69.3 months, which represents late-onset infection, while the mean time from symptom onset to definite diagnosis was 33.8 months. Late-onset PJI is most often due to hematogenous spread [65]. These infections may present with acute onset of systemic symptoms related to bacteremia (similar to early-onset infection), such as pyrexia, and are also characterized by local symptoms, namely joint pain, warmth, tenderness, erythema, edema at the incision site and joint effusion [65]. However, it is not always clear that the infection has been hematogenous and often develops with mild symptoms and, as a result, diagnosis could be delayed [46,66].

As far as the responsible fungal organisms in the present study, the most frequent was *Candida albicans*, reported in in 44 cases (49.4%), followed by *C. parapsilosis* in 16 (18%), *C. glabrata* in 12 (12.4 %), *C. tropicalis* in 5 (5.6%), *C. famata* in 3 (3.3%) and *Cryptococcus neoformans* in 2 (2.2%), while *Pseudallescheria boydii*, *C lipolytica*, *Aspergillus* spp., *C. guilliermondii*, *C. lusitaniae*, *Alternaria infectoria*, *Rhodotorula minuta*, *Pithomyces*, *Aureobasidium*, *Hormonema* and *Coccidioidess* spp. were cultured in 1 case each (1.2%). Co-infection was present in 32 cases (36%), with the most common microorganism being *Staphylococcus* spp. (16 cases; 50%), while other types of organisms have also been isolated. It is notable that coexisting bacterial infection has been mentioned in approximately 15–20% of fungal PJIs [67,68,69]. Bacteria and fungi are thought to act synergistically within the prosthetic biofilm to produce more virulent infections [67]. Patients with more than two co-infective and, especially, multidrug-resistant organisms, had increased risk of recurrent infection that should be considered for appropriate treatment and prognosis [67].

Regarding imaging methods indicating diagnosis, in most patients of the study (20.2%), plain X-ray or CT scans were performed. Despite the fact that radiographic imaging may be considerably valuable, definitive diagnosis cannot be established only by these methods [9,57]. Plain radiographs should be carried out at the onset of suspected PJI so that prosthetic loosening and/or fracture can be seen. Imaging findings are associated with the duration of infection, while 3–6 months are usually required before any manifestation of radiological changes. In addition, low sensitivity as well as specificity for definite PJI diagnosis is reported, as these changes are also observed with aseptic processes [57].

Definite diagnosis may be established through periprosthetic tissue and/or cultures and histopathology. In particular, in 88 of the present cases (98.9%), fungal species were cultured. In 40 (44.9%), fungal PJI was diagnosed through histopathology (38%), while serology testing was additionally performed in 1 case (1.1%). It is of note that serology alone cannot support species identification. Moreover, in 40 (38%) cases, fungal PJI was diagnosed through both histopathology and cultures.

The initial diagnostic algorithm for potential PJI includes plain radiography and measurement of serum inflammatory markers (e.g., ESR and CRP) [57]. Thereafter, a diagnostic arthrocentesis may be performed. [9,68]. Regarding intraoperative specimens, at least three periprosthetic tissue samples (ideally five) should be obtained with different instruments, so that absence of cross-contamination between specimens may be guaranteed. Both cultures and histology examination of intraoperative samples should be performed [57].

Regarding surgical management, TSRA was the most commonly used procedure (47; 52.8%), with a mean time between stages of 7.9 months (SD = 12.5), followed by resection arthroplasty (RA) (19; 21.3%), OSRA (12; 13.5%) and debridement (10; 11.2%), while 5 cases had no surgical treatment (4.5%). Although conclusive guidelines for treatment of fungal PJIs are not established, it appears that TSRA consists of the most preferred surgical intervention [11,12,14]. Other surgical interventions include debridement and retention of prosthesis, OSRA, RA with no reimplantation, arthrodesis or amputation [9,11,12,47,55]. However, it should be noted that both amputation and arthrodesis may severely affect a patient’s quality of life, while OSRA has doubtful outcomes as far as bacterial PJIs are concerned [69,70]. It is worth mentioning that TSRA’s success rate was 92%, while ORSA, RA and debridement exhibited success rates of 92%, 75% and 17%, respectively (Table 3). Revision reconstruction procedures are technically challenging and are frequently associated with inferior outcomes when compared to primary THA. Revision procedures have been associated with higher post-operative complications such as aseptic loosening, periprosthetic fractures, infection, dislocation, and more difficult rehabilitation [70].

Regarding AFT, 48 cases (53.9%) were treated with a single antifungal agent, 33 (37.1%) with two, either simultaneously or consecutively, and 10 (11.2%) with more than two antifungal agents. The mean duration of AFT was found to be 5.1 months. The duration of AFT usually varies and is based on clinical and laboratory findings of each case, as well as physicians’ experience with these types of infections [11,60,68]. Susceptibility testing should be thoroughly performed so that precise MIC values can be obtained, following the isolation of the causative fungus, due to the fact that numerous species of fungi are characterized by resistance to particular antifungal drugs [11].

Fluconazole was the preferred agent in most cases (56; 62.9%), followed by amphotericin B (32; 36%), caspofungin (18; 20.2%), flucytosine (9; 10.1%), voriconazole (6; 6.7%) and micafungin (2; 2.2%), while ketoconazole, anidulafungin, posaconazole and miconazole were used in 1 case each (1.1%). Fluconazole was widely administered in the studied cases, in spite of its inefficacy against molds [68]. However, it should be mentioned that both fluconazole and amphotericin B deoxycholate were the only AFTs available in the early years of the reviewed cases [71,72]. In particular, liver function should be regularly kept under observation during extended fluconazole treatment, as hepatotoxicity has been associated with this agent’s administration [71]. Moreover, amphotericin B is relatively nephrotoxic, limiting its prolonged use. Nonetheless, nephrotoxicity was notably minimized with the use of liposomal amphotericin B compared to the conventional deoxycholate formulation [72,73]. Caspofungin, an echinocandin, is broadly used for invasive candidiasis, particularly in critically ill patients and/or patients with neutropenia [14]. The main benefit of echinocandins compared to other antifungal agents is their fungicidal effectiveness against *Candida* spp., including fluconazole-resistant *C. glabrata* and *C. krusei*, as well as their low renal/hepatic toxicity and drug–drug interactions [14].

More specifically, regarding *Candida* PJIs, which are the most common ones (83 *Candida* spp. were cultured in 79 cases (88.8%)), fluconazole was the preferred agent in 54 cases (68.%, in 26 (48.1%) as monotherapy), followed by amphotericin B in 26 (32.9%, in 11 (42.3%) as monotherapy), caspofungin in 17 (21.5%, 1 (5.9%) as monotherapy), flucytosine in 9 (10.1%, none as monotherapy), voriconazole in 5 cases (6.3%, in 1 (20%) as monotherapy) and micafungin in 2 cases (2.2%, 1 (50%) as monotherapy), while ketoconazole, anidulafungin and miconazole were used in 1 case each (1.1%). The majority of *C. albicans* isolates are susceptible to both fluconazole and echinocandins. Nevertheless, in cases of *C. glabrata*, fluconazole should not be initially applied, since these species are considered fluconazole-resistant. Echinocandins could be the primary AFT for osteoarticular infections due to *C. glabrata*, followed by step-down therapy with oral azoles based on the susceptibilities [46,69].

It is of note that the type and duration of AFT remains controversial. The Infectious Diseases Society of America in cases of fungal septic arthritis recommends either 400 mg fluconazole daily for 6 weeks or an echinocandin, such as caspofungin, for 2 weeks followed by 400 mg fluconazole for at least 4 weeks, while the lipid formulation of amphotericin B for 2 weeks followed by fluconazole for at least 4 weeks represents an alternative option [74]. Nevertheless, in cases that prosthetic implants are present, such as the studied cases, very limited data exist. It has been reported that if the prosthesis may not be removed, chronic suppression could be performed with fluconazole. Regarding fluconazole, the absence of serious adverse effects and favorable pharmacokinetic features of rapid oral absorption with high bioavailability, extended half-life allowing once-daily administration and high concentration of this antifungal in joint fluid approximating that in plasma make it a good choice for the treatment of fungal PJI, especially from an orthopedic point of view [74,75]. It should also be noted that evidence of synergy among antifungals is not clear; however, the antagonistic effect between the agents in use has not been reported so far.

Regarding AAMR in cement, 17 cases (39.5%) were treated with a single agent, 13 (30.2%) with two and 16 (37.2%) with more than two, while in 43 cases (48.3%) no data were available. Vancomycin and amphotericin B were the agents mostly used (60.5% and 51.2%, respectively), followed by gentamycin (51.2%). Cement spacers with antimicrobial agents are recommended in cases of TSRA due to PJI [76,77]. The release of antibiotics from bone cement usually follows a biphasic pattern, with high early release in the first 24 h from the surface of the spacer, then followed by gradual release over the following days. The elution of the antibiotics from bone cement in high concentrations is an important step in healing [76,77]. Based on in vitro studies, only fluconazole, voriconazole and amphotericin B have been evaluated and, thus, may be used for impregnation of bone cement. Literature data are scarce regarding the use of antifungal-impregnated cement in the management of fungal PJIs [75]. A few cases provide information that different antifungal agents can be locally released under in vivo circumstances. However, the ideal impregnation type and dose of antifungals has so far not been defined. It is of note that the type, amount and ratio of the used agent(s), the type and porosity of cement, the surface characteristics and the way the cement is prepared, as well as the environmental circumstances, represent factors affecting the elution kinetics of bone cement. Future studies are needed to investigate the optimal impregnation of bone cement with antifungal agents and evaluate its clinical use in larger samples [75].

During the 2000–2022 study period, the infection’s outcome was successful in 68 cases (76.4%), while the mortality rate was 2.2%. It is notable, however, that the success rate drops to 61.8% in the cases of bacterial co-infection. Hence, bacterial co-infection in fungal hip PJIs should be considered a serious factor increasing the morbidity of the infection.

It is of note that PJIs occurred in 49 (55.1%) surgically revised hip cases (at least one previous surgery), with a mean age of 68.3 years (SD = 14.1). Regarding outcomes of both medical and surgical management, successful rates were reported in 40 cases (81.6%), while the mortality rate was 4% (2 cases).

The present review has some limitations. Not all information from the published cases was available. For example, dosages, drug serum levels, MICs and side effects of the used antifungal drugs, in the vast majority of the cases, were not reported. Nevertheless, this study provides valuable information about epidemiology, symptomatology, diagnosis and medical and surgical management, as well as outcome of cases of fungal hip PJIs.

## 5. Conclusions

Both diagnosis and management of fungal PJIs in patients having undergone THA are quite demanding. A multidisciplinary approach is of utmost importance, since the combination of AFT and TSRA appears to be the proper treatment method. TSRA poses a notable challenge, since great bone defects may exist, while precise preoperative planning is crucial. Based on the fact that outcomes of treatment procedures and policies remain inconclusive, further research and data analysis are required, so that optimal treatment be established.

## Figures and Tables

**Table 1:** Patients’ demographics, causative fungus, bacterial co-infection, comorbidities, number of previous revisions in the same joint, reason of the previous revision, time (T) interval from joint implantation to symptom onset and from symptom to diagnosis are presented. COPD: chronic obstructive pulmonary disease, SLE: systemic lupus erythematosus, DM: diabetes mellitus, (−): not mentioned in the original cases.

Case No	Year	Author	Gender/Age	Fungus	Co-Infection	Immunosuppressive Medication and Conditions	Number of Previous Revisions	Reason of the Previous Revision	T from Implantation to Symptomatology (Months)	T from Symptom Onset to Diagnosis (Months)
1	2001	Ramamohan et al. [15]	F/65	*C. glabrata*	*−*	−	1	Aseptic loosening	48	−
2	2001	Merrer et al. [16]	F/81	*C. albicans*	*−*	DM, Colonic carcinoma	−	−	144	−
3	2001	Marra et al. [17]	M/59	*C. albicans*	*−*	−	2	Aseptic loosening	84	−
4	2001	Bruce et al. [18]	F/68	*C. albicans*	*−*	−	−	−	24	−
5	2001	Bruce et al. [18]	F/51	*C. parapsilosis*	*−*	−	−	−	72	36
6	2002	Phelanet al. [19]	F/75	*C. albicans*	*−*	−	>2	Infection	32.7	27.6
7	2002	Phelanet al. [19]	F/60	*C. albicans*	*−*	−	1	Infection	14.9	10.1
8	2002	Phelanet al. [19]	M/83	*C. albicans*	*−*	−	−	−	184.2	179.6
9	2002	Phelanet al. [19]	M/44	*C. albicans*	*−*	Rheumatoid arthritis	2	−	10	−
10	2002	Phelanet al. [19]	F/75	*C. parapsilosis*	*−*	−	−	−	60.1	36
11	2002	Cutrona et al. [20]	NR/NR	*Rhodotorula minuta*	*−*	−	−	−	−	−
12	2004	Lazzarini et al. [21]	M/63	*C. albicans*	*−*	Chronic monocytic leukemia	−	Infection	7	−
13	2005	Lejko- Zupanc et al. [22]	M/74	*C. glabrata*	*−*	−	−	Loosening	−	72
14	2008	Antony et al. [23]	F/67	*C. parapsilosis*	*−*	−	−	−	−	−
15	2008	Azam et al. [24]	M/73	*C.* *tropicalis*	*−*	−	−	−	108	−
16	2009	Johannsson B and Cakkaggab JJ [25]	M/84	*Cryptococcus neoformans*	*−*	Untreated chronic lymphocytic leukemia, prostate carcinoma	>1	Infection	108	−
17	2010	Kelesidis T and Tsiodraas S [26]	F/93	*C. albicans*	*−*	Colonic carcinoma DM	−	−	5	−
18	2010	Dutronc et al. [27]	F/85	*C. albicans*	*−*	Uterine cancer	>1	Infection	−	−
19	2010	Dutronc et al. [27]	M/66	*C. parapsilosis*	*−*	Chronic kidney failure	>1	Infection	−	−
20	2010	Dutronc et al. [27]	M/77	*C. parapsilosis*	*−*	−	>1	Infection	5	−
21	2011	Gottesman-Yekutiel et al. [28]	F/66	*Pseudallescheria boydii*	*−*	Rheumatoid arthritis, immunosuppressive therapy with steroids	−	−	24	12
22	2012	Bartalesi et al. [29]	F/60	*C. glabrata*	*−*	−	−	−	28	−
23	2012	Anagnostakos et al. [30]	M/78	*C. glabrata*	*−*	Myelodysplastic syndrome	9	Infection	−	−
24	2012	Anagnostakos et al. [30]	F/51	*C. albicans C. glabrata*	*−*	COPD Hepatitis B	6	Infection	−	−
25	2012	Anagnostakos et al. [30]	M/77	*C. lipolytica*	*−*	DM, alcohol consumption, COPD	6	Infection	−	−
26	2012	Anagnostakos et al. [30]	F/68	*C. albicans*	*−*	−	11	Infection	−	−
27	2012	Hall et al. [31]	F/60	*C. glabrata*	*P aeroginosa, E. coli*	Rheumatoid arthritis, vasculitis	−	Infection	−	−
28	2013	Ueng et al. [32]	M/75	*C. albicans*	*−*	Chronic renal insufficiency, COPD	−	−	47	−
29	2013	Ueng et al. [32]	M/64	*C. tropicalis*	*−*	DM, chronic renal insufficiency, COPD	−	−	16	−
30	2013	Ueng et al. [32]	F/62	*C. albicans*	*−*	−	−	−	2	−
31	2013	Ueng et al. [32]	M/66	*C. parapsilosis*	*−*	−	−	−	3	−
32	2013	Lidder et al. [33]	F/76	*C. tropicalis*	*−*	−	1	Aseptic loosening	36	−
33	2013	Artiaco et al. [34]	F/70	*C. albicans*	*−*	Rheumatoid arthritis, Sjogren’s syndrome, immunosuppressive therapy with steroids and methotrexate	3	Infection	72	−
34	2013	Kuiper et al. [35]	M/58	*C. parapsilosis*	*−*	−	−	−	−	−
35	2013	Chiu et al. [36]	M/71	*C. parapsilosis*	*−*	−	−	−	−	48
36	2014	Shah et al. [37]	F/77	*Cryptococcus neoformans*	Gram−negative bacilli	DM, myasthenia gravis, immunosuppressive medication prednisone	−	−	−	−
37	2014	Zhu et al. [38]	M/44	*C. parapsilosis*	*−*	−	−	−	2	−
38	2014	Klatte et al. [39]	M/67	*C. albicans*	*Streptococcus* spp.	DM	7	Infection	6	−
39	2014	Klatte et al. [39]	F/78	*C. albicans*	*−*	DM	4	Infection	1	−
40	2014	Klatte et al. [39]	F/81	*C. glabrata*	*−*	−	3	Infection	8	−
41	2014	Klatte et al. [39]	M/88	*C. albicans*	MRSA, *Ser. Marcescens*	DM, COPD	4	Infection	1	−
42	2014	Klatte et al. [39]	F/62	*C. albicans*	Unspecified bacteria	DM	3	Infection	26	−
43	2014	Klatte et al. [39]	M/31	*C. albicans*	*Streptococcus* spp.	DM	4	Infection	4	−
44	2016	Carrega et al. [40]	F/68	*C. albicans*	*−*	DM	1	periprosthetic fracture	6	−
45	2016	Jenny et al. [41]	F/78	*C. albicans*	*S. epidermis, Propionibacterium acnes*	Hypertension, slight renal Impairment	1	Infection	48	−
46	2017	Cobo et al. [42]	M/77	*C. glabrata*	*−*	Rheumatoid arthritis, immunosuppressive therapy, chronic liver disease from alcohol consumption	3	Loosening, infection	101	7
47	2017	Sebastian et al. [43]	M/53	*C.* *tropicalis*	*−*	−	−	−	24	0
48	2017	Bartash et al. [44]	M/54	*Aspergillus terreus*	*−*	DM, obesity, hepatitis C	3	Infection	13.3	3.5
49	2018	Yong et al. [45]	M/88	*C. albicans*	MRSA, *Ser. Marcescens*	DM, COPD	4	−	4	−
50	2018	Yong et al. [45]	M/66	*C. parapsilosis*	MRSA	COPD	2	−	4	−
51	2018	Yong et al. [45]	M/31	*C. albicans*	MRSA	DM, renal cell carcinoma	1	−	26	−
52	2018	Brown et al. [46]	F/77	*Pithomyces*	*−*	−	−	−	−	−
53	2018	Brown et al. [46]	F/84	*C. albicans*	*−*	−	−	−	−	−
54	2018	Brown et al. [46]	F/75	*C. albicans*	*−*	−	−	−	−	−
55	2018	Brown et al. [46]	M/60	*Aureobasidium,* *Hormonema*	*−*	−	−	−	−	−
56	2018	Brown et al. [46]	M/68	*C. albicans*	*−*	−	−	−	−	−
57	2018	Brown et al. [46]	M/37	*C. albicans*	*−*	−	−	−	−	−
58	2018	Brown et al. [46]	F/63	*C. albicans,*	*−*	−	−	−	−	−
59	2018	Brown et al. [46]	M/89	*Coccidioides* *immitis*	*−*	−	−	−	−	−
60	2018	Brown et al. [46]	F/56	*C. albicans*		−	−	−	−	−
61	2018	Brown et al. [46]	F/75	*C. parapsilosis*		−	−	−	−	−
62	2018	Brown et al. [46]	F/61	*C. glabrata*		−	−	−	−	−
63	2018	Brown et al. [46]	M/45	*C. albicans*		−	−	−	−	−
64	2018	Brown et al. [46]	M/76	*C. albicans*	MRSA	−	−	−	−	−
65	2018	Gao et al. [47]	F/62	*C. tropicalis*	*S. epidermitis, E. coli*	−	3	−	−	36
66	2018	Gao et al. [47]	M/42	*C. albicans*	*Acinetobacter lwoffii*	−	2	−	−	10
67	2018	Gao et al. [47]	F/53	*C. albicans*	*S. aureus*	DM	6	−	−	8
68	2018	Gao et al. [47]	F/43	*C. albicans*	*Enterococcus faecalis*	−	4	−	−	84
69	2018	Gao et al. [47]	M/78	*C. glabrata*	Gram negative bacilli	−	4	−	−	1
70	2019	Pasticci et al. [48]	M/71	*C. glabrata*	*−*	DM	−	−	4	−
71	2020	Saconi et al. [49]	F/64	*C. albicans*	*S. aureus*	DM, alcohol consumption	4	−	6	−
72	2020	Saconi et al. [49]	F/61	*C. parapsilosis*	*S. aureus*	Hepatitis C	4	−	32	−
73	2020	Saconi et al. [49]	M/66	*C. parapsilosis*	*−*	DM	−	−	144	−
74	2020	Saconi et al. [49]	M/63	*C. lusitaniae*	*−*	DM, kidney transplantation, prednisone use	4	−	61	−
75	2020	Saconi et al. [49]	F/53	*C. albicans*	*−*	SLE, methotrexate use	−	−	702	−
76	2020	Saconi et al. [49]	M/61	*C. albicans*	*S. haemolyticus,* *Enterococcus faecium,* *P. aeruginosa*	−	4	−	519	
77	2021	Baecker et al. [50]	NR/85	*C. famata*	*S. epidermidis*	−	2	−	−	−
78	2021	Baecker et al. [50]	NR/81	*C. albicans*	*−*	−	3	−	−	−
79	2021	Baecker et al. [50]	NR/67	*C. famata*	*Citrobacter koser*	−	3	−	−	−
80	2021	Baecker et al. [50]	NR/81	*C. parapsilosis*	*S. caprae*	−	1	−	−	−
81	2021	Baecker et al. [50]	NR/78	*C. parapsilosis*	*−*	−	1	−	−	−
82	2021	Baecker et al. [50]	NR/81	*C. famata*	*−*	−	5	−	−	−
83	2021	Baecker et al. [50]	NR/82	*C. albicans*	*−*	−	7	−	−	−
84	2021	Baecker et al. [50]	NR/78	*C. albicans*	*S. epidermidis*	−	3	−	−	−
85	2021	Baecker et al. [50]	NR/60	*C. parapsilosis*	*Streptococcus sanguinis*	−	7	−	−	−
86	2021	Baecker et al. [50]	NR/83	*Alternaria infectoria*	*−*	−	3	−	−	−
87	2021	Baecker et al. [50]	NR/57	*C. albicans*	*−*	−	6	−	−	−
88	2021	Bottagisio et al. [51]	F/75	*C. albicans*	*S. capitis*	−	2	Infection	372	−
89	2021	Lin et al. [52]	F/76	*C. albicans*	*S. hominis*	Hypertension	3	Infection	9	3

**Table 2:** Definite diagnosis of periprosthetic joint infections caused by fungus and imaging techniques that each case underwent during the process of diagnosing the infection. MRI: magnetic resonance imaging, CT: computer tomography, (−): not mentioned in the original cases, (+): performed in the original cases.

Case	MRI	C/T X-ray	Bone Scanning	Cultures	Biopsy	Serology
1	−	+	−	Joint fluid, tissue specimen	Tissue specimen	−
2	−	+	−	Joint fluid	−	−
3	−	−	−	Joint fluid, tissue specimen	−	−
4	−	−	−	Joint fluid	−	−
5	−	−	−	Tissue specimen	Tissue specimen	−
6	−	−	+	Tissue specimen	−	−
7	−	−	+	Tissue specimen	−	−
8	−	−	+	Tissue specimen	−	−
9	−	−	−	Tissue specimen	−	−
10	−	−	−	Tissue specimen	−	−
11	−	−	−	Tissue specimen	−	−
12	−	−	−	Joint fluid	−	+
13	−	+	−	Tissue specimen	−	−
14	−	−	−	Tissue specimen	−	−
15	−	−	−	Tissue specimen	−	−
16	−	−	+	Joint fluid	−	−
17	−	−	+	Tissue specimen	−	−
18	−	−	−	Tissue specimen	−	−
19	−	−	−	Tissue specimen	−	−
20	−	−	−	Tissue specimen	−	−
21	−	−	+	Joint fluid	−	−
22	−	−	−	Joint fluid	−	−
23	−	+	−	Joint fluid	Tissue specimen	−
24	−	+	−	Joint fluid	Tissue specimen	−
25	−	+	−	Joint fluid	Tissue specimen	−
26	−	+	−	Joint fluid	Tissue specimen	−
27	+	−	+	Joint fluid, tissue specimen	Tissue specimen	−
28	−	−	−	Joint fluid, tissue specimen	Tissue specimen	−
29	−	−	−	Joint fluid, tissue specimen	Tissue specimen	−
30	−	−	−	Joint fluid, tissue specimen	Tissue specimen	−
31	−	−	−	Joint fluid, tissue specimen	Tissue specimen	−
32	−	+	−	Joint fluid, tissue specimen	Tissue specimen	−
33	−	+	−	Joint fluid	−	−
34	−	−	−	Tissue specimen	−	−
35	−	−	−	Tissue specimen	−	−
36	−	+	−	Joint fluid, tissue specimen	Tissue specimen	−
37	−	+	−	−	−	−
38	−	−	−	Joint fluid, tissue specimen	Tissue specimen	−
39	−	−	−	Joint fluid, tissue specimen	Tissue specimen	−
40	−	−	−	Joint fluid, tissue specimen	Tissue specimen	−
41	−	−	−	Joint fluid, tissue specimen	Tissue specimen	−
42	−	−	−	Joint fluid, tissue specimen	Tissue specimen	−
43	−	−	−	Joint fluid, tissue specimen	Tissue specimen	−
44	−	+	+	tissue specimen	Tissue specimen	−
45	−	−	−	Joint fluid, tissue specimen	−	−
46	−	+	−	Tissue specimen	−	−
47	−	−	−	Tissue specimen	−	−
48	−	+	−	Joint fluid, tissue specimen	Tissue specimen	−
49	−	−	−	Tissue specimen	−	−
50	−	−	−	Tissue specimen	−	−
51	−	−	−	Tissue specimen	−	−
52	−	−	−	Tissue specimen	−	−
53	−	−	−	Tissue specimen	−	−
54	−	−	−	Tissue specimen	−	−
55	−	−	−	Tissue specimen	−	−
56	−	−	−	Tissue specimen	−	−
57	−	−	−	Tissue specimen	−	−
58	−	−	−	Tissue specimen	−	−
59	−	−	−	Tissue specimen	−	−
60	−	−	−	Tissue specimen	−	−
61	−	−	−	Tissue specimen	−	−
62	−	−	−	Tissue specimen	−	−
63	−	−	−	Tissue specimen	−	−
64	−	−	−	Tissue specimen	−	−
65	−	−	−	Joint fluid, tissue specimen	−	−
66	−	−	−	Joint fluid, tissue specimen	−	−
67	−	−	−	Joint fluid, tissue specimen	−	−
68	−	−	−	Joint fluid, tissue specimen	−	−
69	−	−	−	Joint fluid, tissue specimen	−	−
70	−	+	−	Joint fluid, tissue specimen	−	−
71	−	−	−	Joint fluid, tissue specimen	Tissue specimen	−
72	−	−	−	Joint fluid, tissue specimen	Tissue specimen	−
73	−	−	−	Joint fluid, tissue specimen	Tissue specimen	−
74	−	−	−	Joint fluid, tissue specimen	Tissue specimen	−
75	−	−	−	Joint fluid, tissue specimen	Tissue specimen	−
76	−	−	−	joint fluid, tissue specimen	Tissue specimen	−
77	−	−	−	Tissue specimen	Tissue specimen	−
78	−	−	−	Tissue specimen	Tissue specimen	−
79	−	−	−	Tissue specimen	Tissue specimen	−
80	−	−	−	Tissue specimen	Tissue specimen	−
81	−	−	−	Tissue specimen	Tissue specimen	−
82	−	−	−	Tissue specimen	Tissue specimen	−
83	−	−	−	Tissue specimen	Tissue specimen	−
84	−	−	−	Tissue specimen	Tissue specimen	−
85	−	−	−	Tissue specimen	Tissue specimen	−−
86	−	−	−	Tissue specimen	Tissue specimen	−
87	−	−	−	Tissue specimen	Tissue specimen	−
88	−	+	−	Tissue specimen	Tissue specimen	−
89	−	+	−	Joint fluid, tissue specimen	Tissue specimen	−

**Table 3:** Surgical and antifungal treatment, duration of AFT, follow-up and infection outcome of the reported cases. ST: surgical treatment, TSRA: two-stage revision arthroplasty, OSRA: one-stage revision arthroplasty, AFT: antifungal treatment, NS: no surgery, RA: resection arthroplasty, (−): not mentioned in the original cases.

Case	ST	Time between Stages in TSRA (Months)	Antimicrobial and/or Antifungal Regimen in Cement	Antifungal Treatment (AFT)	Total Duration of AFT (Months)	Follow-Up (Months)	Outcome
1	TSRA	1.5	−	Amphotericin B, 5−Fluocytosine	1.5	24	Success
2	NS	−	−	Fluconazole	10	11	Success
3	RA	−	−	Fluconazole	1.5	−	−
4	TSRA	3	Fluconazole	Fluconazole	−	48	Success
5	TSRA	11	Fluconazole	Fluconazole	−	84	Success
6	TSRA	12.1	Fluconazole	Fluconazole	2.4	17	Success
7	TSRA	2.4	Fluconazole	Amphotericin B, Fluconazole	11	70	Success
8	TSRA	17.7	−	Amphotericin B, Ketoconazole, Fluconazole	3.5	73	Success
9	TSRA	5	−	Amphotericin B	1.5	60	Success
10	TRSA	14	−	5-Fluorocytosine, Amphotericin B	0.5	24	Success
11	TSRA	12	−	Amphotericin B	−	−	Success
12	RA	−	−	Amphotericin B	1	48	Success
13	RA	−	−	Amphotericin B, Fluconazole, Caspofungin	1	36	Success
14	TSRA	−	−	Fluconazole	−	−	Success
15	TSRA	−	−	Caspofungin, Fluconazole	>3	12	Success
16	RA	−	−	Amphotericin B, Fluconazole	4.3	−	Failure
17	NS	−	−	Fluconazole	>13	12	Success
18	Debridement + RA	−	−	Fluconazole, 5-Fluorocytocine	5	−	Success
19	TSRA	−	−	Fluconazole	6	−	Success
20	RA	−	−	Amphotericin B, 5-Fluocytosine, Fluconazole	9.5	−	Success
21	TSRA	8	Itraconazole	Voriconazole	10	48	Success
22	TSRA	−	−	Voriconazole, Caspofungin, Amphotericin B	2.4	48	Success
23	TSRA	3	Gentamicin, Vancomycin	Fluconazole	1.5	15	Success
24	TSRA	3.5	Gentamicin, Vancomycin	Fluconazole	1.5	70	Success
25	TSRA	3	Gentamicin, Vancomycin	Fluconazole	1.5	22	Success
26	TSRA	3.5	Gentamicin, Vancomycin	Caspofungin	1.5	28	Success
27	RA	−	−	Caspofungin	1.5	−	Success
28	RA	−	Vancomycin, Aztreonam	Fluconazole	−	−	Failure/Death
29	RA	−	Vancomycin, Aztreonam	Fluconazole	−	−	Success
30	RA	−	Vancomycin, Aztreonam	Fluconazole	−	−	Success
31	TSRA	−	Amphotericin, Vancomycin	Fluconazole	−	−	Success
32	TSRA	13	Gentamicin	Amphotericin	6	24	Success
33	Debridement	−	−	Fluconazole, Miconazole	6	12	Failure
34	RA	−	−	Fluconazole	−	2	Failure
35	RA	−	−	Fluconazole	10	24	Success
36	TSRA	3		Amphotericin B	1.5	12	Success
37	NS	−	−	Amphotericin B, Voriconazole	1.5	3	Success
38	OSRA	−	Gentamicin, Clindamycin, Vancomycin	Fluconazole	−	Mean 84	Success
39	OSRA	−	Gentamicin, Clindamycin, Vancomycin	Fluconazole, Amphotericin B	−	Mean 84	Success
40	OSRA	−	Gentamicin, Clindamycin	Flucytosine, Amphotericin B	−	Mean 84	Success
41	OSRA	−	Gentamicin, Clindamycin, Vancomycin	Flucytosine, Amphotericin B	−	Mean 84	Success
42	OSRA	−	Gentamicin, Clindamycin	Voriconazole	−	Mean 84	Success
43	OSRA	−	Gentamicin, Clindamycin, Vancomycin	Flucytosine, Amphotericin B	−	Mean 84	Success
44	TSRA	−	−	Anidulafungin	3	48	Success
45	OSRA	−	−	Voriconazole, Flucytosine. Fluconazole	3	36	Success
46	OSRA	−	−	Caspofungin, Fluconazole	6	5	Success
47	TSRA	−	−	Fluconazole	7	−	Success
48	TSRA	−	Vancomycin, Voriconazole	Posaconazole	−	19.2	Success
49	OSRA	−	−	5-Flucytocine, Amphotericin B	−	>36	Success
50	RA	−	−	Fluconazole, Vancomycin	1	>28	Success
51	TSRA	−	−	Vancomycin, Fluconazole	0.5	−	Failure/Death
52	Debridement	−	Amphotericin B	Amphotericin B	−	−	Success
53	Debridement	−	Amphotericin B	Amphotericin B	−	−	Failure
54	TSRA	NR	Amphotericin B	Amphotericin B	−	−	Success
55	NS	−	−	Amphotericin B	−	−	Success
56	TSRA	NR	Amphotericin B	Amphotericin B	−	−	Failure
57	Debridement	−	Amphotericin B	Amphotericin B	−	−	Failure
58	Debridement	−	Amphotericin B	Amphotericin B	−	−	Failure
59	NS	−	−	Amphotericin B	−	−	Success
60	RA	−	Amphotericin B	Amphotericin B	−	−	Success
61	RA	−	Amphotericin B	Amphotericin B	−	−	Failure
62	RA	−	Amphotericin B	Amphotericin B	−	−	Failure
63	TSRA	NR	Amphotericin B	Amphotericin B	−	−	Failure
64	Debridement	−	Amphotericin B	Amphotericin B	−	−	Failure
65	TSRA	NR	Fluconazole, Vancomycin	Fluconazole, Itraconazole	22	118	Failure
66	TSRA	12	Vancomycin	Fluconazole	4	75	Success
67	TSRA	14	Vancomycin	Fluconazole	4	118	Success
68	TSRA	18	Vancomycin, Meropenem	Fluconazole	4	97	Success
69	TSRA	72	Vancomycin	Fluconazole, Voriconazole, Caspofungin,	11	71	Failure
70	TSRA	8	−	Micafungin	1.8	24	Success
71	Debridement + RA	−	−	Fluconazole	6	−	−
72	OSRA	−	−	Fluconazole	6	84	Success
73	OSRA	−	−	Fluconazole	6	6	Failure
74	OSRA	−	−	Micafungin, Fluconazole	6	−	−
75	Debridement + RA	−	−	Fluconazole	6	71	Success
76	Debridement + RA	−	−	Fluconazole	0.8	74	Success
77	TSRA	1.5	Amphotericin B, Vancomycin, Gentamycin	Caspofungin, Fluconazole	6	44	Failure
78	TSRA	1.5	Amphotericin B, Vancomycin, Gentamycin	Caspofungin, Fluconazole	6	42	Success
79	TSRA	1.5	Amphotericin B, Vancomycin, Gentamycin	Caspofungin, Fluconazole	6	27	Success
80	TSRA	1.5	Amphotericin B, Vancomycin, Gentamycin	Caspofungin, Fluconazole	6	41	Success
81	TSRA	1.5	Amphotericin B, Vancomycin, Gentamycin	Caspofungin, Fluconazole	6	35	Success
82	TSRA	1.5	Amphotericin B, Vancomycin, Gentamycin	Caspofungin, Fluconazole	6	26	Success
83	TSRA	1.5	Amphotericin B, Vancomycin, Gentamycin	Caspofungin, Fluconazole	6	26	Failure
84	TSRA	1.5	Amphotericin B, Vancomycin, Gentamycin	Caspofungin, Fluconazole	6	26	Success
85	TSRA	1.5	Amphotericin B, Vancomycin, Gentamycin	Caspofungin, Fluconazole	6	39	Success
86	TSRA	1.5	Amphotericin B, Vancomycin, Gentamycin	Caspofungin, Fluconazole	6	37	Success
87	TSRA	1.5	Amphotericin B, Vancomycin, Gentamycin	Caspofungin, Fluconazole	6	35	Success
88	TSRA	3	Gentamicin, Clindamycin, Voriconazole	Fluconazole	6	12	Success
89	Debridement + TSRA	−	Vancomycin	Fluconazole	6	12	Success

## Data Availability

Not applicable.
